# Evaluating Causal Links Between Chlorophyll *a* and Environmental Data in the Illinois River (USA)

**DOI:** 10.1002/ece3.73414

**Published:** 2026-04-13

**Authors:** James H. Larson, Lynn A. Bartsch, Kathi Jo Jankowski, Jennifer C. Murphy, Rebecca M. Kreiling

**Affiliations:** ^1^ U.S. Geological Survey Upper Midwest Environmental Sciences Center La Crosse Wisconsin USA; ^2^ U.S. Geological Survey Central Midwest Water Science Center DeKalb Illinois USA

## Abstract

Chlorophyll *a* is an important pigment used by algae to absorb solar energy for photosynthesis. Because chlorophyll *a* is used by all algae (including cyanobacteria) for photosynthesis, it is often measured as an index of algal abundance. Although chlorophyll *a* is an imperfect representation of algal abundance, other methods for quantifying algal abundance are time consuming, expensive, and still imperfect. Chlorophyll *a* can be quantified using a probe that can be deployed for weeks or months at a time, generating high frequency chlorophyll *a* data alongside other water quality data collected with deployed sensors. These other water quality metrics include measurements of several variables that could represent hypothesized drivers of variation in algae abundance. Here, we used water quality and chlorophyll *a* data compiled for the purpose of predicting harmful algal blooms in the Illinois River to identify the strength of relationships between chlorophyll *a* concentration in the water column and other water quality variables that may have a mechanistic link to algal abundance. We used statistical models and causal modeling to evaluate the relationships between environmental data and chlorophyll *a* concentration. We found the highest concentrations of chlorophyll *a* occurred when discharge was low and temperature was high. Relationships were weak to moderate in all modeling approaches that related environmental data to chlorophyll *a* concentration, even when accounting for optimized lag times. The direction and magnitude of statistical associations between chlorophyll *a* and environmental data also varied by site. From a causal modeling perspective, the available data may poorly represent the hypothesized mechanisms, or we may be missing causal drivers of variation in chlorophyll *a* concentration.

## Graphical abstract

Chlorophyll a is widely used as a proxy for algal abundance due to its universality among algae and the practicality of measuring it with in situ sensors. In this study, we analyzed high‐frequency chlorophyll a and water quality data from the Illinois River to assess relationships between algal abundance and environmental drivers using both statistical and causal modeling approaches. While chlorophyll a concentrations were highest during low discharge and high temperatures, overall relationships with environmental variables were weak to moderate and varied by site, suggesting limitations in the data or missing causal factors.

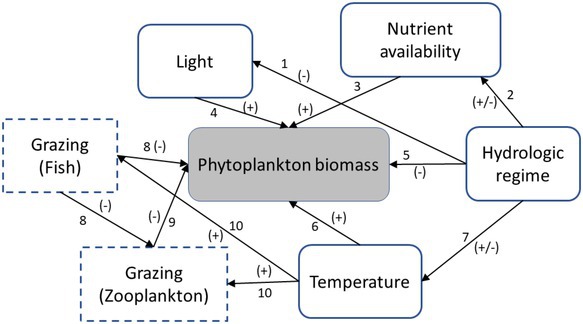

## 1 Introduction

Freshwater eutrophication is considered a major threat to freshwater ecosystems globally (Smith and Schindler 2009; Glibert 2017). Eutrophication refers to very high rates of primary production and is usually associated with excess nitrogen (N) and phosphorus (P; Heisler et al. 2008). In freshwater ecosystems, highly eutrophic lakes often become dominated by cyanobacteria, some of which have a competitive advantage over eukaryotic algae due to their ability to acquire N from the atmosphere (N‐fixation) when P availability increases (Schindler 2012). Other cyanobacteria (e.g., *Microcystis*) are also excellent competitors for P when it becomes limiting (Oliver et al. 2012; Šejnohová and Maršálek 2012). Many common cyanobacteria have the potential to produce cyanotoxins, which can be harmful to humans and aquatic life (Metcalf and Codd 2012; Bownik 2016). When algal biomass is particularly high, dissolved oxygen can be depleted overnight, or when algal cells die, causing hypoxia (Glibert 2017; Blaszczak et al. 2023). As a result, there is interest in identifying environmental conditions that can be used to predict and monitor phytoplankton abundance to minimize risks and remediate ecosystems suffering from eutrophication.

**FIGURE 1:**
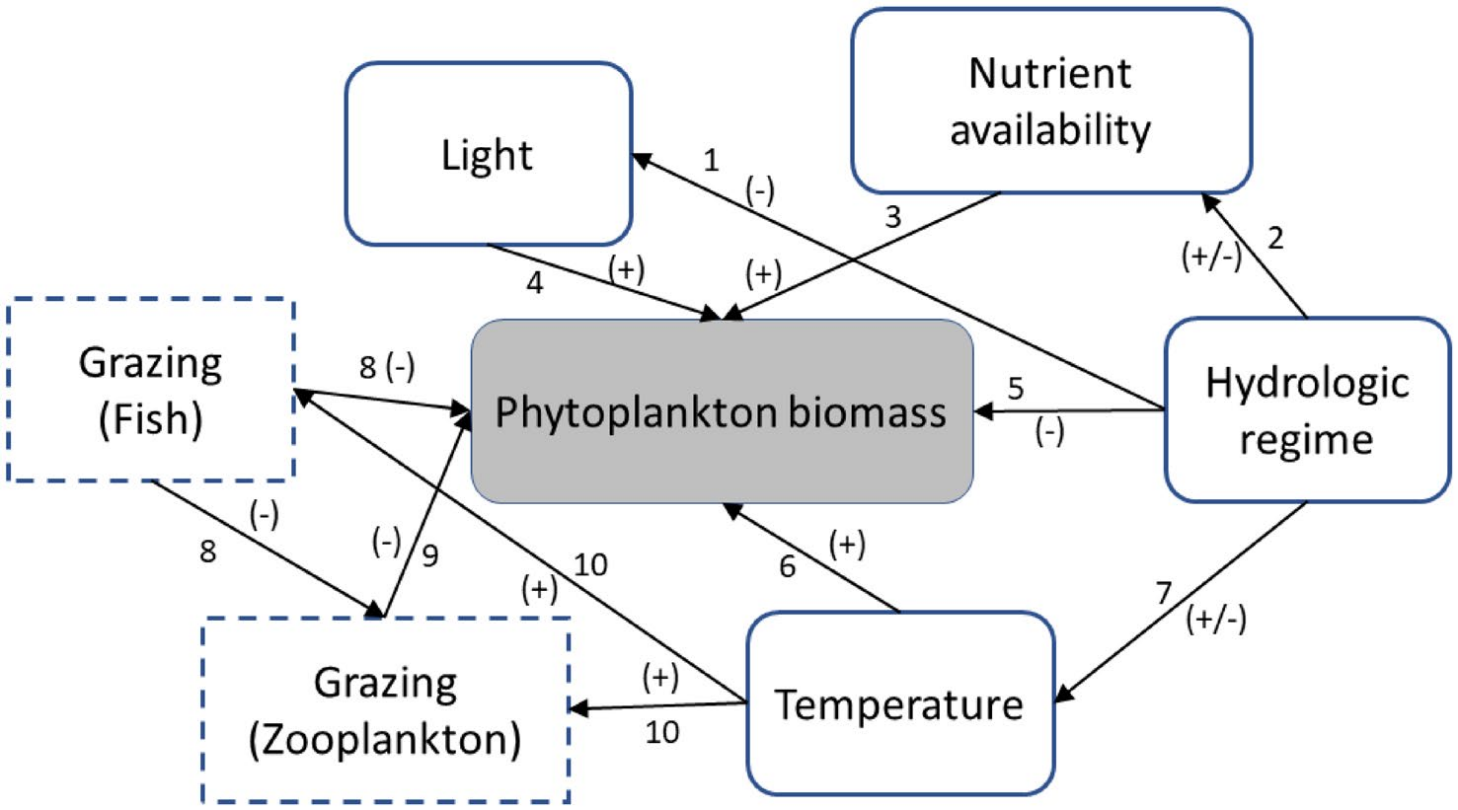
Meta‐model of factors driving variation of phytoplankton biomass in large rivers. Arrow numbers refer to hypothesized effects described in Table 1. The components identified in dashed boxes are those for which data are limited and relationships were not evaluated as part of this study.

Much of the research on the large‐scale control of eutrophication and harmful algal blooms has occurred in large lakes that were initially oligotrophic and then became eutrophic following nutrient additions (Schindler and Fee 1974; Schindler 2012). Less research has been done in riverine systems, but there is still an extensive body of literature that has identified hypotheses related to the drivers of algal abundance (Figure 1; Table 1; Hilton et al. 2006; Xia et al. 2019; Savoy and Harvey 2023; Stackpoole et al. 2024). Nutrients are one of the key drivers of phytoplankton biomass in lakes and are one of several factors hypothesized to drive variation in phytoplankton biomass in rivers as well (Table 1; Soballe and Kimmel 1987; Cha et al. 2017; Xia et al. 2020). Temperature, light availability and grazing pressure are also thought to be highly important in most aquatic ecosystems (Table 1; Figure 1; Cha et al. 2017; Xia et al. 2019; Giblin et al. 2022). However, the distinguishing difference between lakes and rivers is the hydrologic regime: Riverine water residence times are on the order of hours to days, compared to months to years in many lakes. Because hydrology is a factor controlling many aspects of river and stream ecosystems (Reynolds and Descy 1996), both direct and indirect effects of hydrology on phytoplankton production are considered. When discharge increases, phytoplankton accumulation can be outpaced by downstream flushing (Lucas et al. 2009; Nietch et al. 2022; Su et al. 2022), and suspended sediment concentrations often increase (reducing light availability; Delfino 1977; Theiling et al. 1996). Discharge also drives patterns in nutrient concentrations and loads, often in ways that are site specific due to the balance of point and non‐point sources (Jarvie et al. 2006; Bowes et al. 2008). In some river and stream systems, discharge or other hydrologic factors have some influence on temperature, although this is not always a strong relationship (Gray et al. 2018; Giblin et al. 2022). Based on these hypothesized drivers (Table 1), we developed a meta‐model using six key drivers and highlight their direct and indirect effects on phytoplankton biomass (sensu Grace et al. 2010; Grace 2022; Figure 1). Our meta‐model includes grazing pressure from fish and invertebrate grazers, but these data can be difficult to obtain at appropriate time‐scales and were not available for this study.

**TABLE 1:** Hypothesized drivers of phytoplankton biomass and harmful algal blooms.

Arrow	Hypothesis	Citations
1	Discharge influences the light environment experienced by phytoplankton by increasing suspended solids and turbidity which decreases light penetration	Delfino (1977), Soballe and Kimmel (1987), Julian et al. (2008)
2	Increasing river discharge generally dilutes nutrients coming from point sources and increases concentrations from non‐point sources; increasing discharge also increases the river's capacity to carry suspended solids, which carry large amounts of critical nutrients	Delfino (1977), Baker and Baker (1979), Houser and Richardson (2010), Decker et al. (2015)
3	Many elements are used for phytoplankton growth, and influence both the overall productivity of the phytoplankton and the community composition (e.g., nitrogen [N], phosphorus [P], silicon [Si] and iron [Fe]). N and P are often identified as the critical nutrients supporting eutrophication in freshwater ecosystems. Increasing nutrient availability is associated with eutrophication in many aquatic ecosystems	Soballe and Kimmel (1987), Koch et al. (2004), Keck and Lepori (2012), Decker et al. (2015), Wurtsbaugh et al. (2019)
4	Light is a requirement for phytoplankton growth and is often growth‐limiting in large rivers due to high turbidity and high suspended sediment loads	Soballe and Kimmel (1987), Koch et al. (2004), Hilton et al. (2006), Bernhardt et al. (2022)
5	Discharge influences phytoplankton biomass directly by increasing the flushing rate and decreasing residence times, preventing phytoplankton biomass from accumulating	Baker and Baker (1979), Koch et al. (2004), Kim et al. (2020), Sarkar and Kumar (2024)
6	Warmer temperatures are often associated with higher biomass during the growing season. Temperature directly accelerates metabolic processes and alters the solubility of key gases (oxygen and inorganic carbon) in freshwater systems	Paerl and Huisman (2008), Lurling et al. (2013), Wang et al. (2021)
7	Discharge affects temperature by influencing the ratio of surface water and groundwater inputs and by influencing flushing rates and the opportunity for solar heating	Webb et al. (2003), Caissie (2006)
8	Many larval and small fish predate on zooplankton, suppressing large individuals and reducing zooplankton grazing on algae. Other taxa graze on phytoplankton and zooplankton as adults, for example in the Illinois River, gizzard shad ( *Dorosoma cepedianum* ), paddlefish ( *Polyodon spathula* ) and bigmouth buffalo ( *Ictiobus cyprinellus* ) are all planktonic grazers. More recently, the invasive silver carp ( *Hypophthalmichthys molitrix* ) and bighead carp ( *Hypophthalmichthys nobilis* ) have become abundant, which reach high densities and graze on both zooplankton and phytoplankton	Drenner et al. (1982), Yako et al. (1996), Xie (2001), Radke and Kahl (2002), Yang et al. (2006), Calkins et al. (2012)
9	Zooplankton grazers can reduce phytoplankton biomass via direct grazing and can alter the composition of communities by selective grazing	Lehman and Sandgren (1985), Dionisio Pires et al. (2007), Gobler et al. (2007), Davis et al. (2012)
10	Temperature directly influences metabolic rates in a wide variety of animals, strongly influencing growth and secondary production	Gillooly et al. (2002), Chezik et al. (2014), Patrick et al. (2019)

*Note:* Arrow numbers refer to the arrows in the causal diagram from Figure 1.

Monitoring phytoplankton abundance is challenging. For many years, the most common method has involved collecting particulate matter from the water column onto filters, extracting the chlorophyll *a* (chl *a*) pigments using various solvents, and measuring the quantity of extracted pigments (APHA 1998). Extraction and quantification of pigments is desirable because all phytoplankton taxa produce chl *a*, and it is directly tied to photosynthesis. This approach has limitations because the chl *a* content per cell or per gram of biomass is highly dependent on the phytoplankton taxa and physiology (Kruskopf and Flynn 2006). However, other methods for quantifying phytoplankton abundance have limitations as well. For example, microscopy can be used to measure cell concentration or biovolume, but it is more expensive, requires advanced expertise, preservation, and significantly longer processing times in most cases. As a result, estimates of chl *a* remain one of the most used metrics for quantifying variation in algal biomass, assessing the eutrophication risk in a system and testing hypotheses about controls over eutrophication in aquatic ecosystems.

In recent years, our ability to monitor chl *a* in river systems has greatly expanded with the prevalence of in situ chl *a* sensors (Zeng and Li 2015). Chl *a* sensors have provided a much higher frequency assessment of chl *a* dynamics in aquatic ecosystems (e.g., Savoy and Harvey 2023) and strengthened our understanding of temporal variation at multiple scales. However, sensors are a further approximation of algal biomass as they are based on fluorescence, which can vary with river conditions, algal taxa and algal physiology (Zeng and Li 2015). Models estimating chl *a* in rivers are still relatively inaccurate for prediction purposes. For example, Savoy and Harvey (2023) used machine learning to model chl *a* and achieved good model fits, but still had relatively high root mean square errors on predictions (RMSE), especially when applying the model to new sites. Lake chl *a* values seem to be easier to predict, but again RMSE is high relative to median chl *a* concentrations (Song 2025). Studies that use machine learning for chl *a* prediction may be able to find the best possible statistical relationships, but for the purpose of improving our understanding of causal relationships they can be difficult to interpret. For example, Savoy and Harvey (2023) found that dissolved oxygen uptake was a good predictor of chl *a* concentration, but high daily changes in dissolved oxygen are caused by high phytoplankton abundance, not the other way around. For this reason, we approached this analysis with a combination of statistical and causal analysis approaches.

Often, models estimating phytoplankton abundance use measurements of phytoplankton biomass and potential drivers taken at the same time, which may be an incomplete way of considering these relationships. Phytoplankton responses will typically lag changes in the environment and can be related to processes occurring over several timescales, on the order of days to weeks (Duarte 1990; Hampton et al. 2017; Wilkinson et al. 2020; Liu et al. 2022). In the case of river systems, temporal lags also correspond to spatial lags and lags in the strength of relationships could be due to these spatial changes as well. This lagged response may occur for several reasons, such as physiological constraints in the ability of phytoplankton to respond to changes (Collos 1986; Davidson and Cunningham 1996) or that environmental measurements are an incomplete representation of the underlying process (e.g., water velocity vs. hydrological regime; Liu et al. 2022). For example, there is a lag between a change in the availability of key resources and the competitive exclusion of taxa that are not suited to that new resource regime. In non‐equilibrium conditions (which are the rule rather than the exception in lotic systems), this lag makes it possible for species co‐existence even when different species have very different growth rates in that nutrient regime (Litchman and Klausmeier 2001). Thus, it is important to understand how lag times may affect the relationship between environmental drivers and phytoplankton production. Sensors provide a means of evaluating these types of lags in a more robust way than discrete monitoring approaches can typically provide due to the logistical costs associated with processing discrete samples.

Accurately monitoring phytoplankton is a major tool for assessing harmful algal blooms (HABs). HABs are a byproduct of eutrophication, where dense accumulations of phytoplankton trigger environmental or economic harms. In freshwaters, the most common HABS‐forming taxa are cyanobacteria. HABs are often difficult to define precisely but are usually characterized by very high chl *a* concentrations, as well as distinctive surface scums, bright green surface waters and the presence of toxins (Gorney et al. 2023). The Illinois River basin is highly developed with urban and suburban land use in the upper portion of the basin, which transitions in the lower basin to predominantly agricultural land (mostly row crops). Due to large inputs of treated wastewater and agricultural runoff, nutrient concentrations are high throughout the basin, and HABs occur sporadically. Many of these hypothesized environmental drivers of phytoplankton abundance can be difficult to measure directly, but surrogate measures are collected routinely with water quality sensors. Platt et al. (2022) compiled a dataset of sensor‐measured chl *a* and water quality parameters for the purpose of understanding the drivers of HABs in the Illinois River basin. Here we used the Platt et al. (2022) dataset to test predictive relationships between phytoplankton abundance and environmental properties (Table 1, Figure 1), using sensor‐derived data. We used a suite of analytical approaches to identify (1) water quality conditions when blooms are most likely to occur, (2) statistical associations between water quality data and chl *a*, and (3) causal interactions among the different hypothesized drivers of phytoplankton abundance.

## 2 Methods

### 2.1 Study Area

**FIGURE 2:**
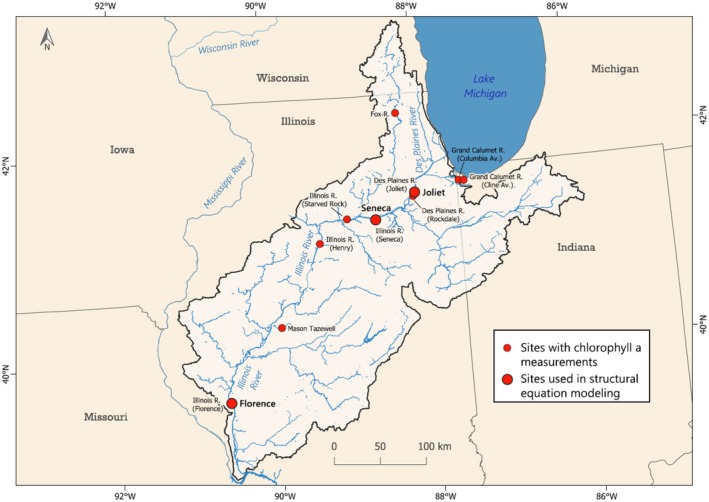
Streams and rivers where chlorophyll *a* concentrations were estimated using fluorescence sensor in the Illinois River basin (2005–2024; Platt et al. 2022). The larger circles labeled Joliet, Seneca and Florence are the sites with the most data on chlorophyll *a* plus other environmental data and were used to parameterize structural equation models to test for causal interactions of hypothesized drivers. Base map for state boundaries from ESRI, U.S. States (Detailed). Base map for rivers modified from U.S. Geological Survey (2024), National Hydrography Dataset.

The Illinois River is a large, floodplain river that connects the Great Lakes at Chicago to the Mississippi River at Grafton, Illinois (Figure 2). The catchment is 72,700 km^2^ and most of the ~439 km of the main stem Illinois River is navigable with the assistance of lock and dam structures and regular dredging. There is a large change in slope downstream of the confluence with the Fox River tributary (near Starved Rock State Park) that divides the river into the upper and lower Illinois River. The river contains a mixture of main channel, side channel, contiguous, and isolated backwater areas that cover a wide range in residence time and hydraulic connectivity to the main river flow. These physical differences set up gradients in water velocity (Houser et al. 2013; DeBoer et al. 2018), temperature (Jankowski et al. 2020), and nutrient concentrations (De Jager and Houser 2012; Carey et al. 2019) that can influence algal productivity and communities.

### 2.2 Sensor‐Based Measurements

Phytoplankton biomass was represented by chl *a* measured using a fluorescence probe (USGS parameter code 32316; “Chlorophyll fluorescence (fChl), water, in situ, concentration estimated from reference material, micrograms per liter as chlorophyll” and parameter code 32318; “Chlorophylls, water, in situ, fluorometric method, excitation at 470 + −15 nm, emission at 685 + −20 nm, micrograms per liter”). Sensor data was compiled into daily means. There were 10 sites with continuous chl *a* data, which included data from 2005 to 2024 (Figure 2, Table 2). All fluorescence measurements included temperature data but only a subset included co‐occurring measurements of other water quality parameters (i.e., nutrients and light). Just three sites included large enough sample sizes (*N* > 1000) of environmental data to parameterize the meta‐model (using structural equation modeling); Figure 1: the Des Plaines River (at Joliet; USGS station number 05537980; 2017–2022), the Illinois River (at Seneca; USGS station number 05543010; 2013–2022) and the Illinois River (at Florence; USGS station number 05586300; 2014–2023). These three sites essentially cover most of the longitudinal gradient of the Illinois River basin, along the main stem (Figure 2).

**TABLE 2:** Sites used in the univariate analysis (all sites) and structural equation modeling (in bold) from the Illinois River basin (Platt et al. 2022).

Site number	Site description	Latitude (deg N)	Longitude (deg E)	Chlorophyll *a* (μg mL^−1^)	Temp/Chlorophyll *a* (N)	Turbidity (N)	NO_X_ (N)	OP (N)	Dis (N)
05536356	Grand Calumet R. (Columbia Av.)	41.6186	−87.4998	9.6 (0.7, 62.8)	387	382	—	—	—
**05537980**	**Des Plaines R. (Joliet)**	**41.5364**	**−88.0825**	**4.8 (0.1, 32.7)**	**1835**	**1835**	**1830**	**1115**	**1835**
05538020	Des Plaines R. (Rockdale)	41.5000	−88.1069	5.3 (0.4, 34.4)	2570	2549	—	—	1752
**05543010**	**Illinois R. (Seneca)**	**41.2997**	**−88.6142**	**5.0 (1.1, 41.4)**	**2934**	**2759**	**2732**	**—**	**2301**
05549500	Fox R.	42.3100	−88.2515	37.4 (2.3, 136.0)	812	812	—	—	393
05553700	Illinois R. (Starved Rock)	41.3247	−88.9840	15.2 (2.5, 68.7)	1587	485	369	—	815
05558300	Illinois R. (Henry)	41.1072	−89.3561	20.8 (4.5, 75.7)	1504	67	—	—	1504
05568705	Mason Tazewell	40.3265	−89.9141	1.3 (0.4, 13.2)	399	399	—	—	—
**05586300**	**Illinois R. (Florence)**	**39.6327**	**−90.6078**	**13.2 (2.7, 56.2)**	**2676**	**2685**	**2387**	**1090**	**1920**
413,646,087,260,101	Grand Calumet R. (Cline Av.)	41.6128	−87.4336	2.0 (1.0, 4.4)	184	—	—	—	2

*Note:* Columns labeled N indicate the number of observations included for the univariate analysis. Sites with no data for a particular predictor variable are designated with a dash (—). The median (with range) of chlorophyll *a* values is reported.

Abbreviations: Dis, discharge; NO_X_, nitrate plus nitrite; OP, orthophosphate; Temp, water temperature.

Other sensor data were used to represent environmental conditions hypothesized to be important for phytoplankton biomass. Temperature was represented by water temperature, which was available whenever fluorescence estimates of chl *a* were recorded (USGS parameter code 00010; “Temperature, water, degrees Celsius”). Nutrient availability was represented by two measurements: nitrate + nitrite (NO_X_; USGS method 99,133; description: “Nitrate plus nitrite, water, in situ, milligrams per liter as nitrogen”) and total inorganic phosphorus (OP; 51289; “orthophosphate, water, in situ, milligrams per liter as phosphorus”). These measurements were not available in many cases when chl *a* was measured. Nutrient availability is hypothesized to influence phytoplankton biomass, but inorganic nutrient concentrations may reflect the “leftover” nutrients available after phytoplankton uptake (as has been observed in other river systems; Houser et al. 2015). Light availability was represented by turbidity (63,680; “Turbidity, water, unfiltered, monochrome near infra‐red LED light, 780‐900 nm, detection angle 90 +‐2.5 degrees, formazin nephelometric units [FNU]”). As with NO_X_ and OP, turbidity was often unavailable when chl *a* was measured. Turbidity is an adequate index of light availability, although phytoplankton abundance itself contributes to turbidity (Giblin et al. 2022). Hydrologic regime was represented by discharge (00060; “Discharge, cubic feet per second”). We converted discharge from cubic feet per second to cubic meters per second. All of these measurements are reported as daily mean values.

### 2.3 Statistical Analysis

We calculated median water temperature, turbidity, NO_X_, OP and discharge when chlorophyll *a* was high (> 95% quantile) or low (<median value) using data from all sites. We compared environmental parameters between the high and low chlorophyll *a* categories using the Mann–Whitney *U*‐test, using a *p*‐value of 0.05 as a threshold indicating the categories were different. Note that with very large sample sizes, as is the case here, *p*‐values almost always have very small values regardless of the ecological significance.

Only three sites had > 3 years of continuous data spread throughout the year and at least 4 relevant environmental parameters: the Des Plaines River at Joliet and the Illinois River at Seneca and Florence. All of the remaining analyses are focused on these three sites. We calculated the average value for chl *a* and discharge for each day of the year (ordinal dates) and plotted these against the annual hydrograph. All statistical analyses were performed using R (R Development Core Team 2022).

Before parameterizing the structural equation model, we investigated the optimal lag times for relationships between individual environmental parameters and chl *a*. This was done using simple linear relationships between chl *a* and environmental parameters with different lag times. For example, we parameterized a linear model where temperature on day *t* was regressed against chl *a* on day *t*. We then repeated this model using temperature on day *t*‐1, *t‐*2…*t‐*14, as well as average temperature during the previous week, 2 weeks or 30 days. For each model relating temperature to chl *a* in this example, we estimated the standardized slope (*β*) and coefficient of determination (*R* ^2^). This was done for all of the relationships identified in the meta‐model (water temperature, turbidity, NO_X_, OP and discharge relationships with chl *a*; discharge relationships with water temperature, turbidity, NO_X_ and OP). Models were generated independently for each site. All variables were log‐transformed prior to this analysis to improve the normality of model residuals. We considered *β* of less than |0.2| to be a weak effect, |0.2–0.4| to be moderate and | > 0.4| to be a large effect (Cohen 1992). The optimal lag time was subsequently used in the structured equation models (SEM), rather than using day‐of measurements.

For each site, an SEM was parameterized using the lavaan package in R (Rosseel 2012), following principles described by Grace (2006) (Grace et al. 2015) and detailed implementation in R described by Lefcheck (2024). In this approach, an initial model structure is compared to models that include additional connections that are either causal relationships or co‐variances, and the final model structure is one that is selected based on model comparisons. Models are compared using Akaike's information criterion (AIC; Burnham and Anderson 1998). When using AIC, lower values are better, and a common rule of thumb is that differences among models (ΔAIC) are considered meaningful when differences are at least > 2. We used AIC corrected for small sample sizes (AIC_C_), which converges to AIC when sample sizes increase. Model fit is assessed using the comparative fit index (CFI) and the chi‐squared test (comparing the fit of a particular model to a saturated model; Grace 2022). In this assessment, a chi‐squared *p* value of > 0.05 is preferred and a CFI of > 0.95 is considered good. Orthophosphate (OP) data are limited in the Platt et al. (2022) dataset. As a result, we left OP out of our structural equation models and included only NO_X_ to represent nutrient availability. Prior to analysis, response and predictor variables were log‐transformed, since log‐transformation appeared to improve model residuals.

Autocorrelation, the correlation among repeated measurements of chl *a* over time, was estimated using R, using the “acf” function. This analysis found that autocorrelation was extremely high. Including a 1‐day lag term reduced this autocorrelation substantially, and therefore we re‐parameterized a structural equation model with a 1‐day lag term included (i.e., chl *a* concentration today is predicted by chl *a* yesterday). To account for autocorrelation in the SEM, we added a 1‐day lag term to the model (e.g., chl *a* on day t‐1 is related to chl *a* on day t).

In addition to assessing autocorrelation with a 1‐day lag term, we also evaluated the statistical relationship between chl *a* concentration and measurements from 1 day before, 5 days before and the average over the previous week. To do this we parameterized either simple linear regressions (with chl *a* as the response variable) or logistical regressions (to identify when chl *a* was above the 95% quantile at that site). To evaluate model performance, we held out 30% of the data and used predictions from these models to estimate the root mean square error (RMSE) and the *R* ^2^ value between observed and predicted. For the logistic regression models, we calculated the Brier score (Brier 1950) and the Nagelkerke *R* ^2^ of the logistic model (Nagelkerke 1991). We also calculated the concentration at which the model predicts a 70% likelihood of chl *a* being greater than the 95% quantile value.

## 3 Results

### 3.1 Water Quality Conditions When Blooms Are Most Likely to Occur

**FIGURE 3:**
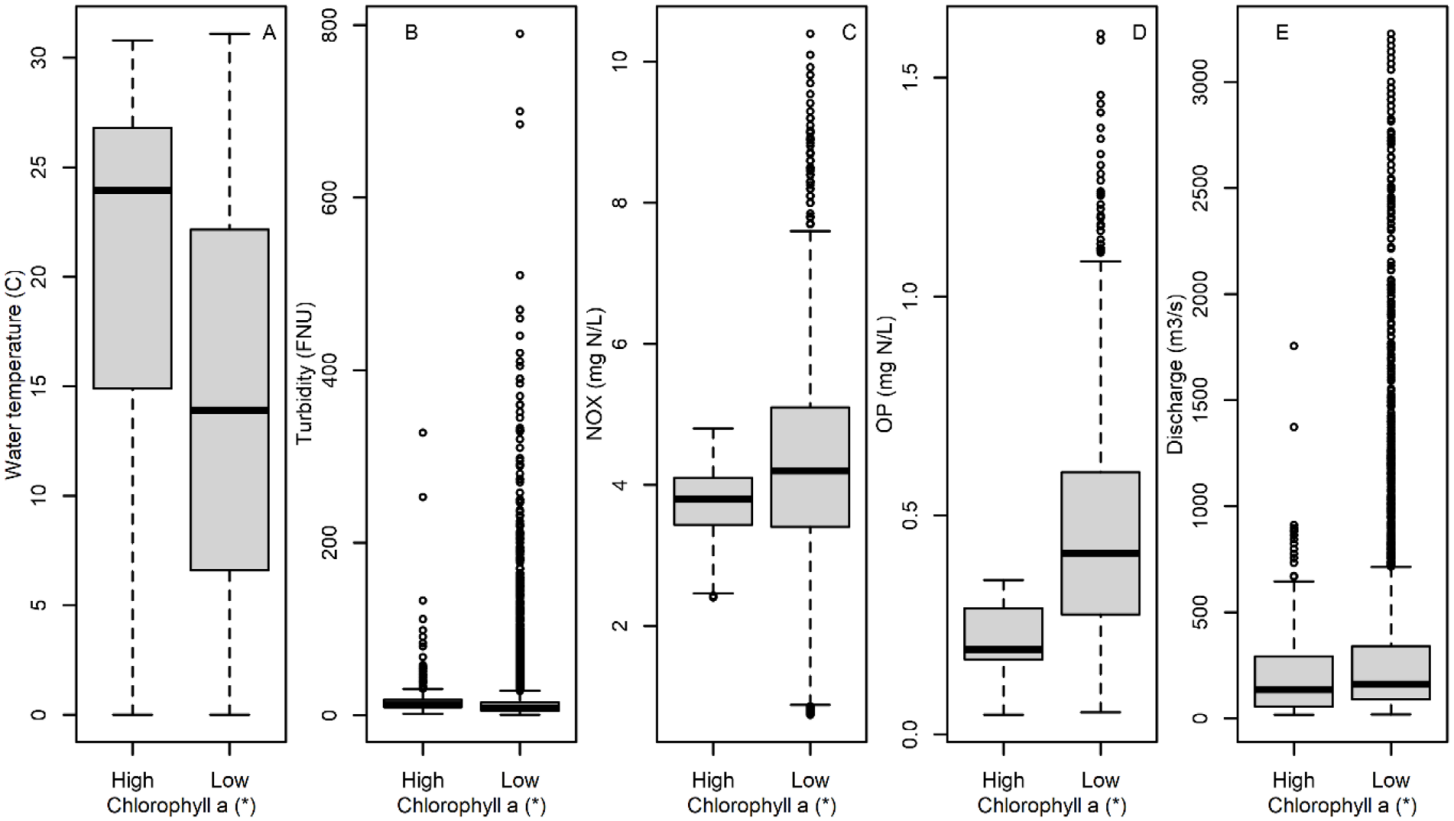
Box‐and‐whisker plots showing environmental parameters (Panels [A] water temperature, [B] turbidity, [C] nitrates plus nitrites [NO_X_], [D] orthophosphate [OP] and [E] discharge) observed at times when chlorophyll *a* was high (≥ 95% quantile) or low (≤ 50% quantile). Mann–Whitney *U*‐test indicate the differences between high and low chlorophyll *a* conditions are all statistically significant (indicated by an *). Boxes encompass the first and third quartiles. The thick black line is the median. The lines (whiskers) show the largest or smallest observation that falls within 1.5 times the box size. Observations that fall outside the lines are shown individually. FNU, formazin nephelometric units.

The median concentration from all sites in the basin was 8.4 μg chl *a* L^−1^ (range 0.1–136.0 μg chl *a* L^−1^), and the 95% quantile was 38.7 μg chl *a* L^−1^. Therefore basin‐wide “Low Chlorophyll” was defined as observations when chl *a* was ≤ 8.4 μg L^−1^ and “High Chlorophyll” was observations when chl *a* was ≥ 38.7 μg L^−1^. Observations with High Chlorophyll tended to have high temperature and turbidity, but low discharge, NO_X_, and OP relative to observations with Low Chlorophyll (Table 3, Figure 3). Of the three sites with the most data (Des Plaines, Illinois River at Seneca, and Illinois River at Florence), only the Illinois River sites ever reached the “High Chlorophyll” category for the basin.

**TABLE 3:** Environmental conditions when chlorophyll *a* is extremely high (≥ 95th quantile) or low (≤ 50th quantile) in the Illinois River basin (Platt et al. 2022; U.S. Geological Survey 2024).

Environmental variable	Median (≥ 95th quantile of chlorophyll *a*)	Median (≤ 50th quantile of chlorophyll *a*)
Temperature* (°C)	24.0 (*N* = 737)	13.9 (*N* = 7385)
Turbidity* (FNU)	11.9 (*N* = 471)	8.4 (*N* = 6962)
Nitrates plus nitrites [NO_X_]* (mg nitrogen per L^−1^)	3.8 (*N* = 59)	4.2 (*N* = 4242)
Orthophosphate [OP]* (mg phosphorous per L^−1^)	0.19 (*N* = 26)	0.41 (*N* = 1237)
Discharge* (m^3^ per s^−1^)	135.2 (*N* = 430)	160.8 (*N* = 5236)

*Note:* Sample sizes are noted. We performed the non‐parametric Mann–Whitney *U*‐test to assess whether the environmental conditions were from equivalent populations.

Abbreviations: FNU, formazin nephelometric units; *N*, number.

* A Mann–Whitney *U*‐test indicated the populations were not equal (*p* < 0.05).

**FIGURE 4:**
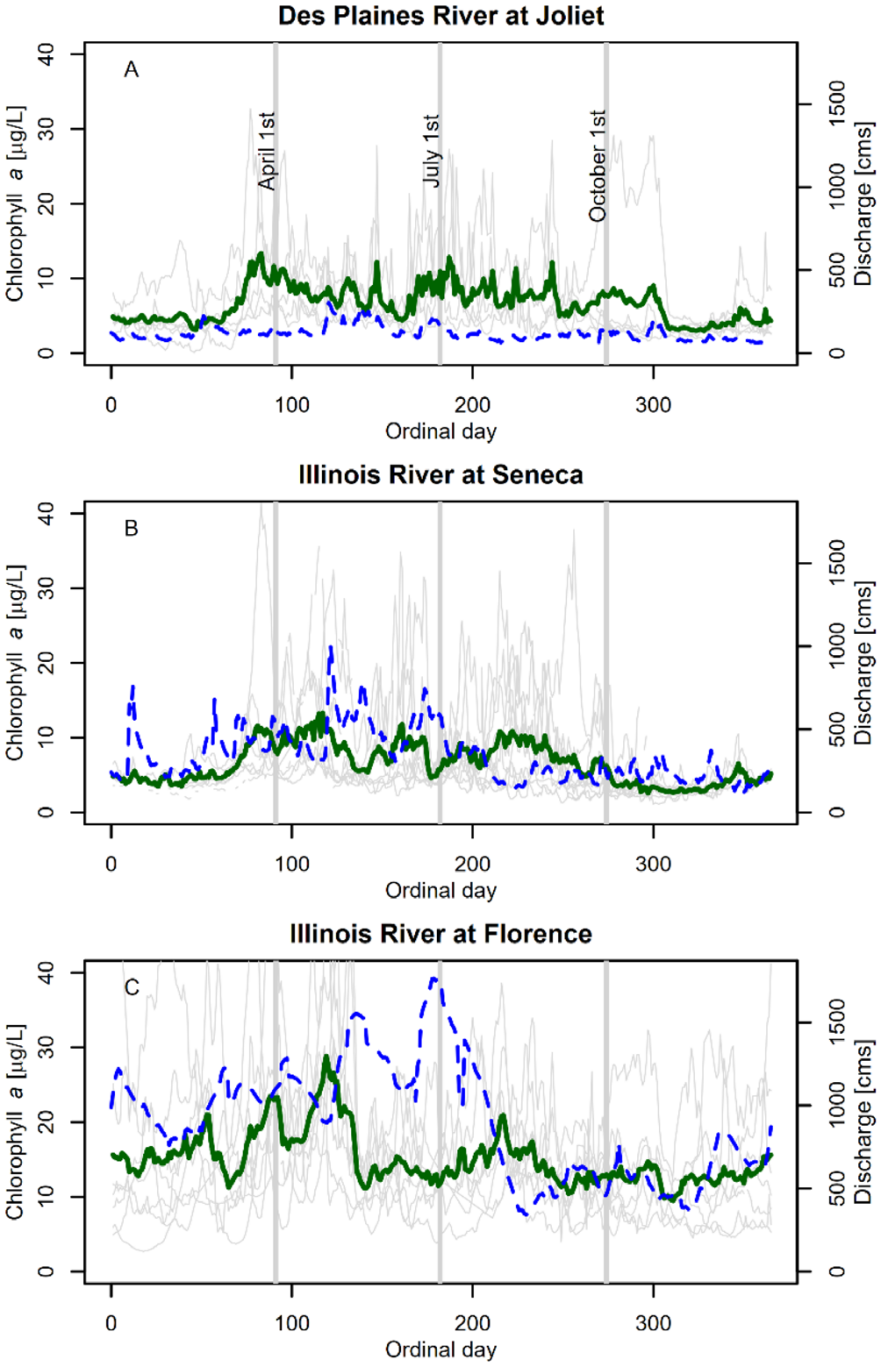
Chlorophyll *a* concentration (left axis, green line) and discharge (right axis, blue dashed line) averaged by ordinal day of the year at three sites in the Illinois River basin (2005–2024; Panel [A] Des Plaines River, [B] Illinois River at Seneca and [C] Illinois River at Florence). Chlorophyll *a* concentrations for each individual year are shown in light gray lines. These are averages of the data available in Platt et al. (2022) and additional data from the U.S. Geological Survey (2024). Monitoring frequency is inconsistent across this time period and remaining analyses are focused on recent years (> 2018).

The three data‐rich sites spanned a spatial gradient from an upstream tributary (Des Plaines River at Joliet; median chl *a* 4.8 μg L^−1^) to the steeper section of the Illinois River (Seneca; 5.0 μg chl *a* L^−1^) to the low gradient conditions in the lower Illinois River (Florence; 13.2 μg chl *a* L^−1^; Figure 4). Median discharge increased from 93 m^3^s^−1^ at the Des Plaines site to 751 m^3^s^−1^ at the most downstream site (Illinois River at Florence; Figure 4). Chl *a* concentrations were high early in the growing season (April–May) and increased again in late summer (Figure 4, Table S1).

### 3.2 Accounting for Lag Time Between Environmental Data and Chl a

We focused on the three sites with the most data for the univariate and SEM analysis (Des Plaines River at Joliet, Illinois River at Seneca, Illinois River at Florence). Site‐specific standardized slopes from univariate models between chl *a* and environmental measurements made on the same day, and averages over the previous week, 2 weeks or previous month (30 days) were similar (Table 4). For example, in the Des Plaines River at Joliet, the standardized slope of water temperature (β_TEMP_) on chl *a* was 0.34 using same day measurements and increased to 0.35, 0.36 and 0.40 when averaged over the prior week, 2 weeks and 30 days, respectively (Table 4). There were some exceptions, however, such as the relationship between chl *a* and turbidity from the Illinois River at Florence, which was negative using measurements collected on the same day, but positive using measurements collected 10 days prior by almost the same absolute value (Table 4). In most cases the strongest effect size from the previous 14 days had a stronger or equivalent relationship to chl *a* as any of the other time lag metrics we modeled (Table 4). In all cases, these univariate models explained very little variation in chl *a* concentration (*R* ^2^ < 0.2; Table 4).

**TABLE 4:** Standardized slope (*β*) of simple linear regression between response and predictor variables integrated over different time periods (same day, over the past week, over the past 2 weeks or over the past 30 days) at 3 sites in the Illinois River basin (2005–2024).

Site	Response	Predictor	*β*—day of	*β*—past week	*β*—past 2 weeks	*β*—past 30 days	*β*—optimal day (from past 14 days)	*R* ^2^—optimal
Des Plaines River Joliet, IL	Chlorophyll	Temperature	0.34	0.35	0.36	0.40	0.38 (14 days prior)	0.14
	Chlorophyll	Turbidity	−0.06	−0.09	−0.13	−0.15	−0.18 (12 days prior)	0.03
	Chlorophyll	NO_X_	−0.08	−0.10	−0.09	−0.08	−0.10 (3 days prior)	0.01
	Chlorophyll	OP	−0.09	−0.10	−0.09	−0.11	−0.10 (3 days prior)	0.01
	Chlorophyll	Discharge	0.13	0.10	0.08	0.11	0.13 (day of)	0.04
	Temperature	Discharge	0.15	0.12	0.06	−0.005	0.15 (day of)	
	Turbidity	Discharge	0.75	0.58	0.46	0.39	0.75 (day of)	
	NO_X_	Discharge	−0.68	−0.55	−0.45	−0.36	−0.68 (day of)	
	OP	Discharge	−0.51	−0.43	−0.37	−0.35	−0.51 (day of)	
Illinois River Seneca, IL	Chlorophyll	Temperature	0.22	0.20	0.21	0.25	0.29 (14 days prior)	0.08
	Chlorophyll	Turbidity	0.06	0.08	0.11	0.13	0.17 (14 days prior)	0.03
	Chlorophyll	NOX	−0.09	−0.05	−0.02	−0.02	−0.09 (day of)	0.01
	Chlorophyll	Discharge	−0.20	−0.21	−0.21	−0.21	−0.20 (day of)	0.04
	Temperature	Discharge	−0.04	−0.12	−0.17	−0.26	−0.15 (14 days prior)	
	Turbidity	Discharge	0.77	0.68	0.62	0.55	0.77 (day of)	
	NO_X_	Discharge	0.17	0.18	0.17	0.23	0.21 (14 days prior)	
Illinois River Florence, IL	Chlorophyll	Temperature	0.01	0.01	0.003	0.002	0.07 (14 days prior)	0.004
	Chlorophyll	Turbidity	−0.13	−0.001	0.07	0.03	0.14 (10 days prior)	0.02
	Chlorophyll	NOX	−0.16	−0.17	−0.16	−0.13	−0.18 (3 days prior)	0.03
	Chlorophyll	OP	−0.15	−0.10	−0.04	0.03	0.15 (14 days prior)	0.02
	Chlorophyll	Discharge	−0.28	−0.28	−0.25	−0.21	−0.29 (2 days prior)	0.09
	Temperature	Discharge	0.03	0.001	−0.04	−0.12	−0.09 (14 days prior)	
	Turbidity	Discharge	0.15	0.18	0.17	0.10	0.17 (4 days prior)	
	NO_X_	Discharge	0.59	0.59	0.59	0.60	0.59 (day of)	
	OP	Discharge	−0.35	−0.33	−0.32	−0.34	−0.35 (day of)	

*Note:* The optimal past day was estimated using plots (e.g., S1) showing the modeled relationship given different potential number of days of lag. In this analysis, 14 days was the longest daily lag period we considered mechanistically plausible for the hypotheses in Table 1. No orthophosphate (OP) data were available for the Illinois River at Seneca.

Abbreviation: NO_X_, nitrates.

### 3.3 Causal Interactions Among the Different Hypothesized Drivers of Phytoplankton Abundance

**FIGURE 5:**
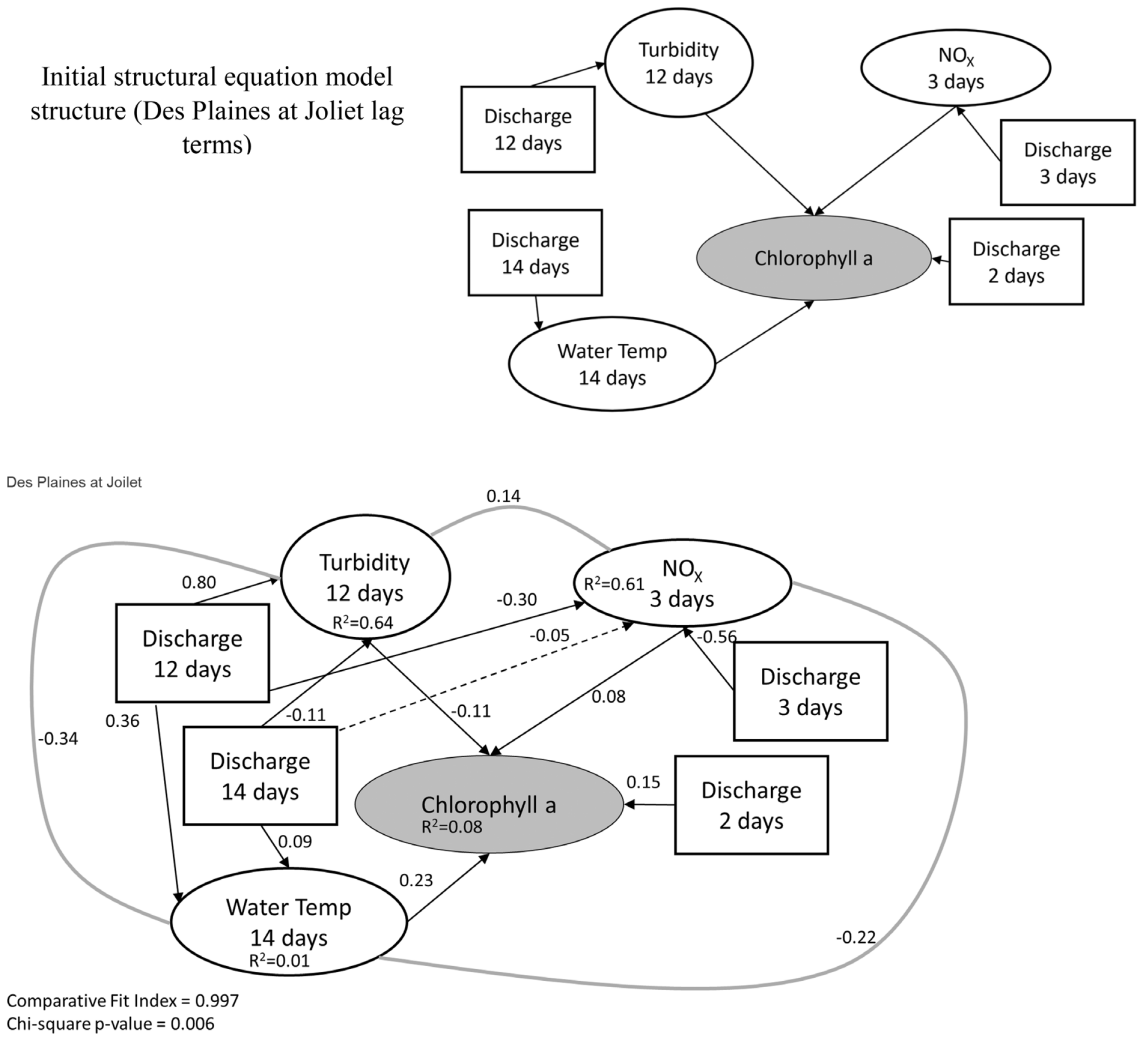
Initial and parameterized structural equation model that is optimized for the lag times identified in Table 5 for the Des Plaines River at Florence (IL, USA). For each relationship, we identified the lag time that resulted in the maximum standardized slope for each relationship and used those data to parameterize the model. Lag times are all relative to the measurement of chlorophyll *a* (e.g., nitrate+nitrite [NO_X_] concentrations from 3 days prior are used and discharge from that same day is used to predict NO_X_). Dashed lines are relationships where effect sizes are indistinguishable from zero, solid black lines are relationships where effect sizes are distinct from zero and gray solid lines are co‐variation between variables that lack a clear mechanistic basis. Numbers on individual lines are standardized coefficients. Square boxes indicate exogenous variables, and elliptical boxes indicate endogenous variables. We have shaded the chlorophyll *a* box, since that is the focus of our hypotheses.

**FIGURE 6:**
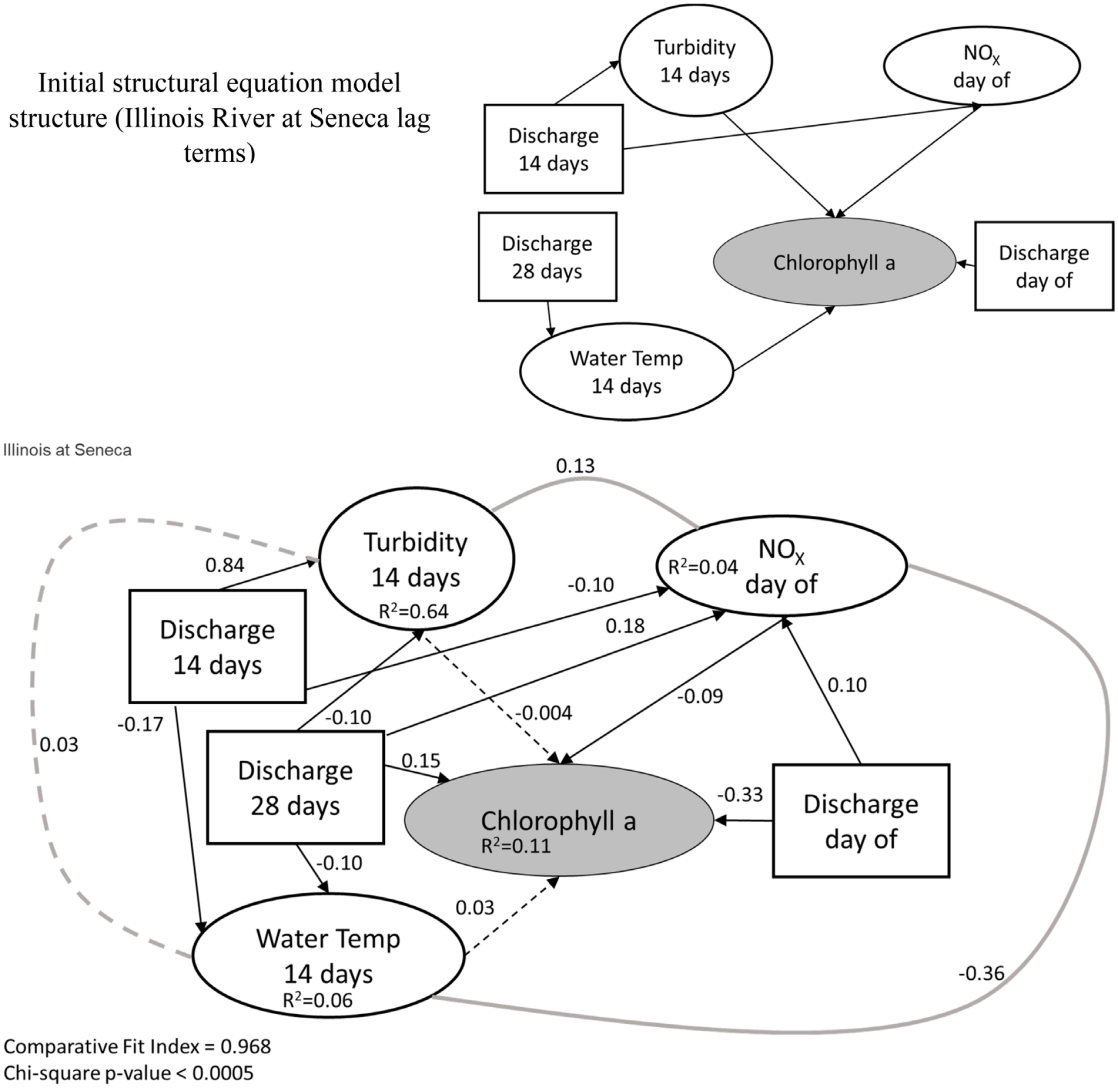
Initial and parameterized structural equation model that is optimized for the lag times identified in Table 5 for the Illinois River at Seneca (IN, USA). For each relationship, we identified the lag time that resulted in the maximum standardized slope for each relationship and used those data to parameterize the model. Lag times are all relative to the measurement of chlorophyll *a* (e.g., turbidity concentrations from 14 days prior are used and discharge from that same day is used to predict turbidity). Dashed lines are relationships where effect sizes are indistinguishable from zero, solid black lines are relationships where effect sizes are distinct from zero and gray solid lines are co‐variation between variables that lack a clear mechanistic basis. Numbers on individual lines are standardized coefficients. Square boxes indicate exogenous variables, and elliptical boxes indicate endogenous variables. We have shaded the chlorophyll *a* box, since that is the focus of our hypotheses. NO_X_ = nitrate + nitrite.

**FIGURE 7:**
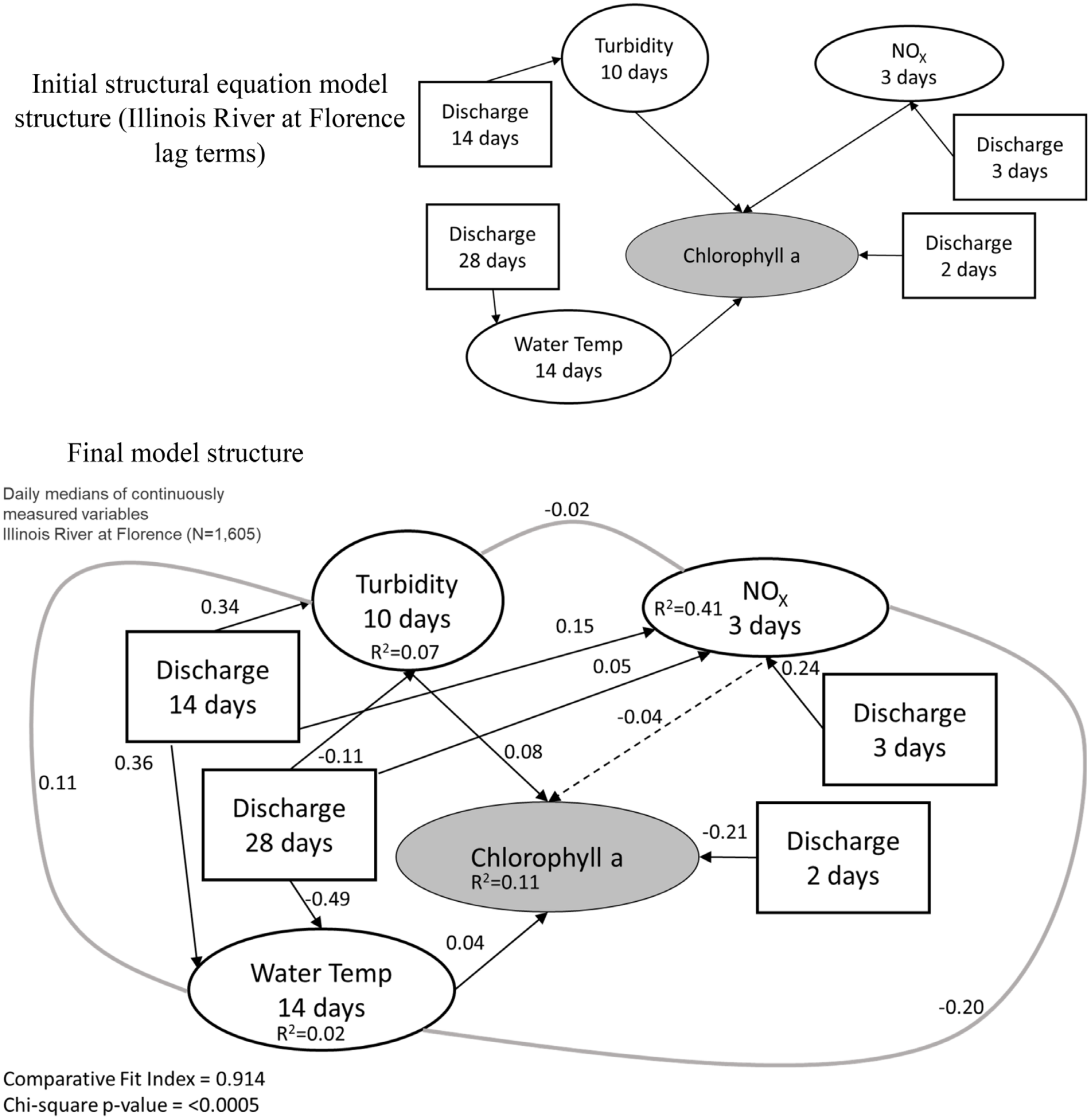
Initial and parameterized structural equation model that is optimized for the lag times identified in Table 5 for the Illinois River at Florence (IN, USA). For each relationship, we identified the lag time that resulted in the maximum standardized slope for each relationship and used those data to parameterize the model. Lag times are all relative to the measurement of chlorophyll *a* (e.g., nitrate+nitrite [NO_X_] concentrations from 3 days prior are used and discharge from that same day is used to predict NO_X_). Dashed lines are relationships where effect sizes are indistinguishable from zero, solid black lines are relationships where effect sizes are distinct from zero and gray solid lines are co‐variation between variables that lack a clear mechanistic basis. Numbers on individual lines are standardized coefficients. Square boxes indicate exogenous variables, and elliptical boxes indicate endogenous variables. We have shaded the chlorophyll *a* box, since that is the focus of our hypotheses.

We translated our meta‐model (Figure 1) into a structural equation model (SEM) for each site using available data (with the optimized lag terms; Table 4) to represent different conceptual components. Phytoplankton biomass was represented by chl *a* concentration, light availability by turbidity, temperature by water temperature, nutrient availability by NO_X_ and hydrologic regime by discharge. Only three sites contained all these data with a large enough sample size to parameterize the resulting SEM: Des Plaines at Joliet, Illinois River at Seneca, and Illinois River at Florence. After parameterizing the model and testing the addition or removal of additional co‐variances (using AIC_C_), the best overall model structure (lowest ΔAIC_C_ by more than 2 AIC_C_ units) for each site had very low *R* ^2^ values (0.09–0.12) for chl *a* (Figures 5, 6, 7). For each site, models included many additional cause‐effect or co‐variance relationships environmental data components of the model. Standardized effect sizes for individual predictors in chl *a* were moderate to weak (< 0.4; Figure 5). For example, water temperature had a moderate positive effect on chl *a* at Des Plaines and the Illinois River at Seneca, but a near‐zero effect in the Illinois River at Florence (Figures 5, 6, 7).

### 3.4 Autocorrelation and Prediction of Chl a at Illinois River (Florence)

**FIGURE 8:**
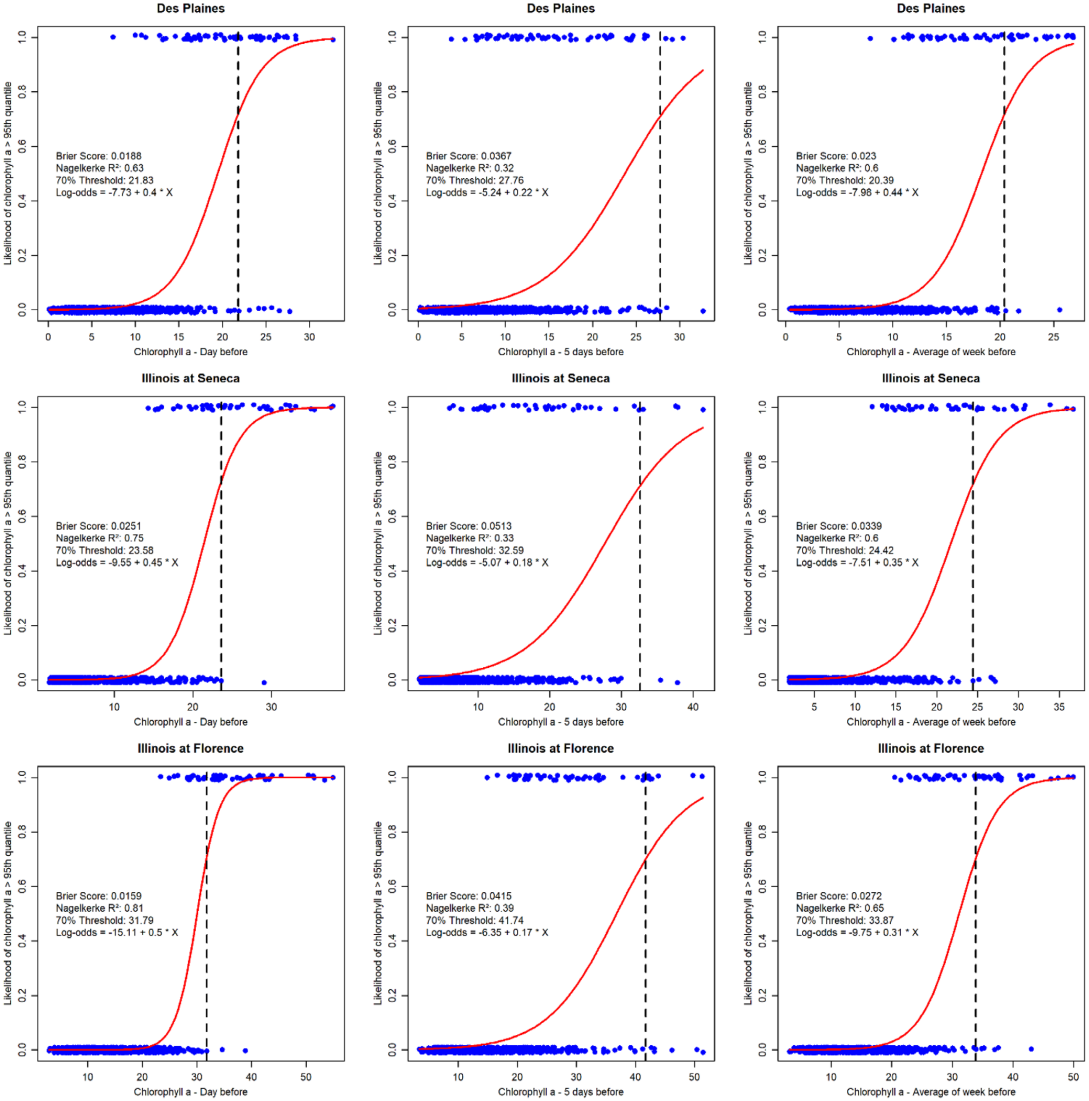
Probability of chlorophyll a being ≥ 95% quantile in relationship to the chlorophyll *a* concentration the day before, 5 days before or the average of the previous week in the Des Plaines River at Joliet, the Illinois River at Seneca, or the Illinois River at Florence (IL, USA). Dots are observed data (with a small jitter to improve visibility). Solid line is a fitted logistic model (equation on the plot). Brier Score and Nagelkerke *R* ^2^ are similar to the root mean square error and coefficient of determination from linear models. The value that yields a 70% chance of chlorophyll *a* being ≥ 95% quantile is identified with a vertical dashed line.

**FIGURE 9:**
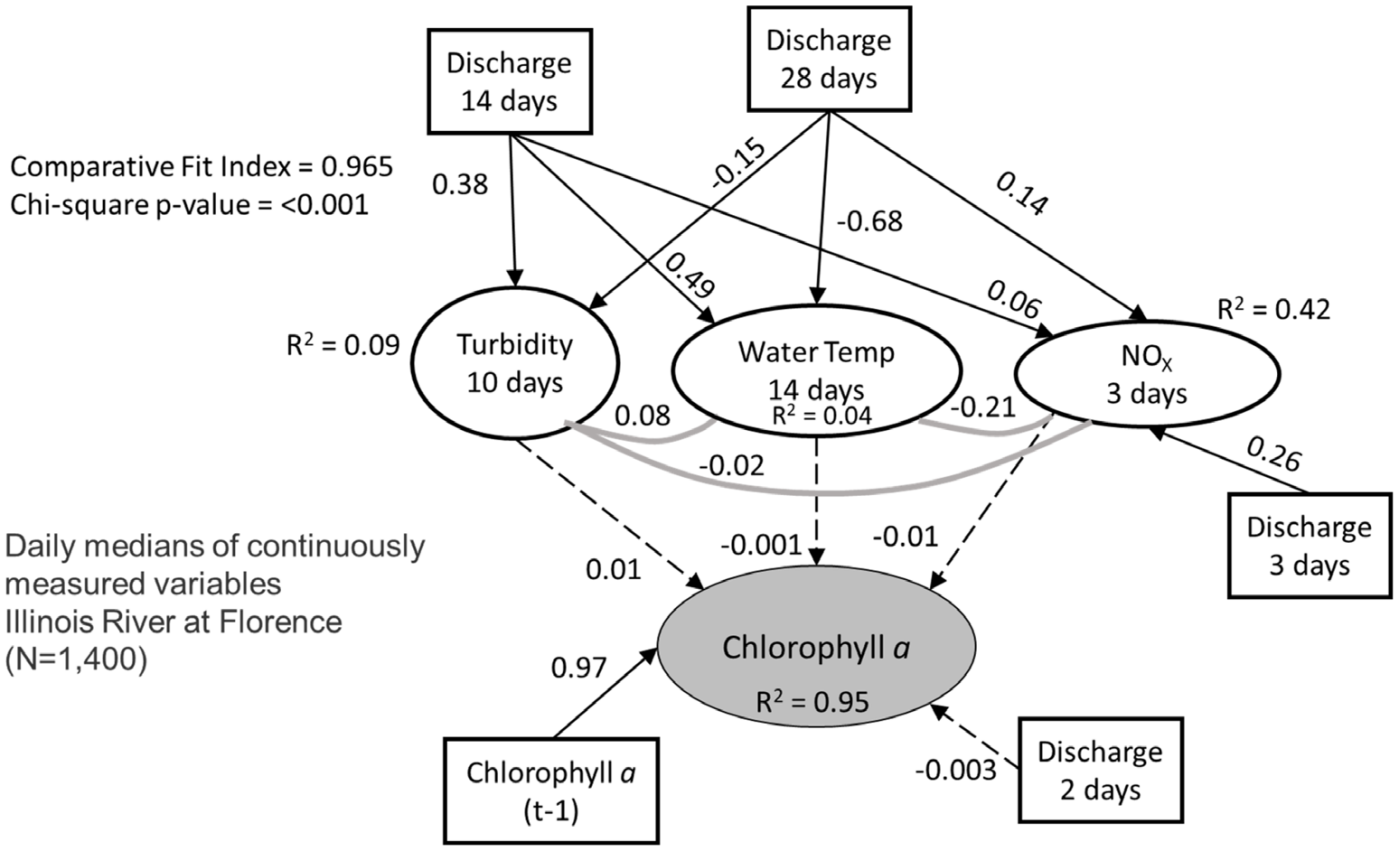
Parameterized structural equation model for the Illinois River at Florence (IL, USA) that is optimized for the lag times identified in Table 5 and a 1 day autocorrelation term for chlorophyll *a* for the Illinois River at Florence. For each relationship, we identified the lag time that resulted in the maximum standardized slope for each relationship and used that data to parameterize the model (Table 5). Lag times are all relative to the measurement of chlorophyll *a* (e.g., nitrate+nitrite [NO_X_] concentrations from 3 days prior are used and discharge from that same day is used to predict NO_X_). Dashed lines are relationships where effect sizes are indistinguishable from zero, solid black lines are relationships where effect sizes are distinct from zero and gray solid lines are co‐variation between variables that lack a clear mechanistic basis. Numbers on individual lines are standardized coefficients. We have shaded the chlorophyll *a* box, since that is the focus of our hypotheses.

The large datasets available from the Des Plaines (Joliet), Illinois River (Seneca), and Illinois River (Florence) consisted of long periods of continuous daily measurements. Daily measurements of chl *a* were highly autocorrelated: Chl *a* on Day 1 had a Pearson's *r* of > 0.95 with chl *a* on Day 2. Linear models relating chl *a* only to chl *a* measurement the day before, 5 days before, or over the previous 7 days all demonstrated much stronger associations than any of the models we considered with environmental data (Table 5, Figure S2). Site specific “High Chlorophyll” values were 16.8, 18.1, and 30.7 μg chl *a* L^−1^ at Des Plaines, Illinois at Seneca, and Illinois at Florence, respectively. Logistic models were good at predicting the likelihood of a day having “High Chlorophyll” (as indicated by Nagelkerke *R* ^2^ and Brier Scores; Figure 8). However, these autocorrelation models mainly indicate day‐to‐day changes in chl *a* are near‐zero on average. For example, logistic models predict a 70% likelihood of High Chlorophyll occurring only when High Chlorophyll is already occurring on earlier days. Incorporating autocorrelation into the SEM analysis results in a model structure that includes links to environmental data, but all the environmental drivers have near‐zero effect sizes (*R* ^2^ = 0.95; See parameterized SEM for Illinois River at Florence in Figure 9, other sites are similar but not shown here).

**TABLE 5:** Statistical association between observed and predicted chlorophyll *a* concentrations in the Illinois River basin (IL, USA) using chlorophyll *a* measured the day before, 5 days before or an average over the previous 7 days as a predictor in a simple linear regression.

Site	*β*_N‐1_	Day before (*R* ^2^/RMSE)	*β*_N‐5_	5 days before (*R* ^2^/RMSE)	*β*_Av7_	7 day average (*R* ^2^/RMSE)
Des Plaines River at Joliet	0.95	0.89/0.32	0.79	0.61/0.61	0.92	0.83/0.39
Illinois River at Seneca	0.96	0.92/0.27	0.79	0.61/0.67	0.91	0.84/0.44
Illinois River at Florence	0.96	0.93/0.25	0.76	0.58/0.62	0.91	0.83/0.40

Abbreviations: *R* ^2^, coefficient of determination; RMSE, root mean squared error.

## 4 Discussion

High chl *a* concentrations in the Illinois River basin were positively associated with warmer temperatures and lower discharge, as we hypothesized, but effect sizes within individual sites were very weak. No univariate models explained even a fifth of the variation in chl *a* concentrations, despite focusing on a single site and seeking the ideal lag time. Combining these univariate parameters into a causal modeling approach (SEM) did not improve the fit between chl *a* and environmental variables. Overall, either our initial meta‐model was missing important causal relationships, or the hypothesized causal links were poorly represented by the data.

There was one factor in our meta‐model that we lacked data to evaluate (grazing). Fish and zooplankton grazer abundance and even grazing rates can be quantified, but typically estimates are done seasonally (especially for fish) because of the effort involved. There are no other major mechanisms that we could identify that were not included in the meta‐model, but there could be unknown mechanisms we have failed to identify. Further, it is possible that we have included all the important covariates but their relationships with chlorophyll either can't be modeled with a linear (or log‐linear) relationship or important drivers of chl *a* change over the season or period of record.

Our data may be insufficient representations of our hypothesized relationships. We used data previously compiled for the purpose of predicting HABs (Platt et al. 2022), but site selection, the parameters, and the sampling intervals are dictated by many different purposes and logistical limitations. In other words, the data were not actually collected for the explicit or sole purpose of HABs monitoring. As a result, the available data may not be an accurate representation of our hypothesized relationships. For example, chl *a* is not really an ideal reflection of phytoplankton biomass or biovolume (Kruskopf and Flynn 2006), and the in situ fluorescence probe is not an ideal method for accurately measuring chl *a* (Kruskopf and Flynn 2006; Liu and Georgakakos 2021). We used nitrate and orthophosphate to characterize nutrient availability, but phytoplankton require 25 elements, and the availability of many of them is either unknown or rarely measured (Kaspari and Powers 2016). Even for N, there are many other forms of dissolved N which are preferentially taken up by certain phytoplankton groups (Donald et al. 2013; Reinl et al. 2022) such as ammonium, urea, metabolic macromolecules, and humic and fulvic acids. Turbidity is an index of light penetration within the water column, but is partially caused by phytoplankton and is not a measure of incident light at the surface. Discharge, a factor in all river systems, may not be the correct way to characterize all aspects of the hydrologic regime. For example, water velocity and water residence times drive accumulation and flushing rates, and the relationship between these and discharge is possibly non‐linear (Giblin et al. 2022).

The limitations in the data available result in limitations in our ability to interpret and predict causal relationships. For example, low concentrations of nitrate can occur either when phytoplankton are absent and incoming loads are low, or could occur when phytoplankton abundance is very high and most of the available nitrate is being taken up into phytoplankton biomass (Houser et al. 2015). Data similar to Platt et al. (2022) are available in many places across the country due to efforts by the U.S. Geological Survey's water quality monitoring programs and other regional and state programs. These sensors (in particular, chl *a*, nitrate and turbidity) record “continuously” (usually every 1 min to 1 h) and are deployed in situ, so large datasets can quickly accumulate. Because prediction of HABs in large riverine systems is an area of active research, there are several efforts to use these water quality measurements to improve chl *a* predictions. However, as demonstrated here, these data were not necessarily sufficient representations of hypothesized relationships.

The only data that we observed with a high statistical and causal connection to daily chlorophyll concentration was chlorophyll concentration on earlier days. This model may not be an accurate method to predict concentration, as it essentially predicts that conditions will continue as they are (i.e., chlorophyll concentration will not change). If the fluorescence sensor used here really is an accurate representation of phytoplankton abundance, then this observation of very strong autocorrelation suggests that the phytoplankton community changes slowly on a day‐to‐day or week‐to‐week basis. Additional sources of data on phytoplankton abundance could be evaluated to confirm the accuracy of the sensor. Similar to other efforts, we demonstrated that the best predictive power came from chl *a* on the day prior, suggesting that intrinsic population dynamics of phytoplankton communities could be considered in these predictive models.

Improving the model performance could be achieved by allowing more flexibility in model structure (beyond linear and log‐linear models) or statistically by adding many more variables with limited consideration for their mechanistic importance. These approaches have been used with some success in other systems (e.g., Francy et al. 2016), including with XGBoosted regressions (Savoy and Harvey 2023; Song et al. 2025). Inclusion of variables without a clear direct causal mechanism linking them to phytoplankton growth may still represent or reveal underlying latent variables that influence phytoplankton growth (e.g., variability in conductivity may be driven by salts associated with fertilizers and developed land uses). However, purely statistical models are conceptually limited to the conditions in which the original model data were collected. New data can be outside the original conditions in ways that are both obvious (e.g., predictors that range beyond the values in the training data) and cryptic (e.g., changes in rainfall patterns, land use, etc.), and in both cases the effect on model accuracy will be unknown. Therefore, causal models (and process models) can be used even when they have less apparent predictive power in the short term. We founded our approach on well‐established and process‐based understanding of phytoplankton communities, which can benefit from improved process‐based understanding of controls of phytoplankton communities in rivers.

## Author Contributions

**James H. Larson:** conceptualization (equal), formal analysis (equal), funding acquisition (equal), investigation (equal), methodology (equal), project administration (equal), supervision (equal), validation (equal), visualization (equal), writing – original draft (equal). **Lynn A. Bartsch:** conceptualization (equal), funding acquisition (equal), investigation (equal), methodology (equal), validation (equal), visualization (equal). **Kathi Jo Jankowski:** conceptualization (equal), formal analysis (equal), investigation (equal), methodology (equal), project administration (equal), resources (equal), validation (equal), visualization (equal), writing – review and editing (equal). **Jennifer C. Murphy:** conceptualization (equal), data curation (equal), formal analysis (equal), funding acquisition (equal), methodology (equal), project administration (equal), resources (equal), validation (equal), visualization (equal), writing – review and editing (equal). **Rebecca M. Kreiling:** conceptualization (equal), formal analysis (equal), investigation (equal), methodology (equal), validation (equal), visualization (equal), writing – review and editing (equal).

## Funding

This work was supported by the U.S. Geological Survey.

## Ethics Statement

The authors have nothing to report.

## Conflicts of Interest

The authors declare no conflicts of interest.

## Supporting information


**Table S1:** Monthly median chlorophyll *a* concentrations at three sites in the Illinois River basin (IL, USA), 2013–2024 (Platt et al. 2022; U.S. Geological Survey 2024). Monitoring frequency is inconsistent across this time period and remaining analyses are focused on recent years (> 2018). All chlorophyll *a* concentrations are in units of μg chl *a* L^−1^.
**Figure S1:** Relationship between the standardized slope of a model relating chlorophyll *a* to turbidity and the lag in that model between the day the chlorophyll *a* was measured and the day the turbidity was measured. For example, the standardized slope of a model relating chlorophyll *a* measured at day zero to the turbidity measured 10 days before is highlighted in the vertical blue line (this is also the lag day with the greatest slope in this example). Solid line is the standardized slopes and dashed lines are the 95% confidence interval around those standardized slope estimate. The numbers are the maximum and minimum sample sizes (fewer samples are available as the lag times increase). To select the optimal lag time, we used this figure to identify the maximum absolute value of the standardized slope.
**Figure S2:** Comparison of model predictions of chlorophyll *a* (μg L^−1^) from univariate linear regression models versus observations that were not included in the original model training. Predictors were log transformed chlorophyll *a* (μg L^−1^) concentration the day before, 5 days before or the average of the previous week in the Des Plaines River at Joliet, the Illinois River at Seneca, or the Illinois River at Florence (IL, USA). Dots are observed data, dashed line is the 1:1 line. The root mean square error (RMSE) and coefficient of determination (*R* ^2^) between observations and predictions is reported on the figure.

## Data Availability

The data used in this paper are a “harmonized” dataset of continuous water quality data compiled for modeling HABs in the Illinois River basin (Platt et al. 2022, doi:10.5066/P9RISQGE). This dataset includes harmonized data from the U.S. Geological Survey's (USGS) Water Data for the Natio, the U.S. Army Corps of Engineers, the Illinois Environmental Protection Agency, and a USGS Open‐File Report. Data from USGS were downloaded using software as described in Platt et al. (2022). The Platt et al. (2022) dataset only included data up to 2020, so we re‐ran the workflow to get additional data from USGS through May 8, 2024 (U.S. Geological Survey 2024).
