# Supplementary material for: Evaluating Causal Links Between Chlorophyll a and Environmental Data in the Illinois River (USA)
Source: Ecol Evol. 2026 Apr 13;16(4):e73414. doi: 10.1002/ece3.73414 (PMC13071481; doi:10.1002/ece3.73414)

Table S1. Monthly median chlorophyll *a* concentrations at three sites in the Illinois River basin (IL, USA), 2013-2024 (Platt et al. 2022, U.S. Geological Survey 2024). Monitoring frequency is inconsistent across this time period and remaining analyses are focused on recent years (>2018). All chlorophyll *a* concentrations are in units of µg chl *a* L^-1^.

| Month | Des Plaines River at Joliet | Illinois River at Seneca | Illinois River at Florence |
| --- | --- | --- | --- |
| January | 4.0 | 4.3 | 11.5 |
| February | 3.5 | 3.9 | 14.9 |
| March | 7.2 | 6.3 | 14.4 |
| April | 7.2 | 7.9 | 19.5 |
| May | 6.8 | 5.2 | 14.2 |
| June | 5.4 | 5.5 | 12.4 |
| July | 7.3 | 6.2 | 13.2 |
| August | 6.6 | 8.4 | 14.8 |
| September | 5.2 | 4.2 | 11.7 |
| October | 3.5 | 3.1 | 11.3 |
| November | 3.3 | 2.9 | 9.9 |
| December | 3.6 | 4.3 | 11.4 |

Figure S1. Relationship between the standardized slope of a model relating chlorophyll *a* to turbidity and the lag in that model between the day the chlorophyll *a* was measured and the day the turbidity was measured. For example, the standardized slope of a model relating chlorophyll *a* measured at day zero to the turbidity measured ten days before is highlighted in the vertical blue line (this is also the lag day with the greatest slope in this example). Solid line is the standardized slopes and dashed lines are the 95% confidence interval around those standardized slope estimate. The numbers are the maximum and minimum sample sizes (fewer samples are available as the lag times increase). To select the optimal lag time, we used this figure to identify the maximum absolute value of the standardized slope.

Figure S2. Comparison of model predictions of chlorophyll *a* (µg L^-1^) from univariate linear regression models versus observations that were not included in the original model training. Predictors were log transformed chlorophyll *a* (µg L^-1^) concentration the day before, 5 days before or the average of the previous week in the Des Plaines River at Joliet, the Illinois River at Seneca, or the Illinois River at Florence (IL, USA). Dots are observed data, dashed line is the 1:1 line. The root mean square error (RMSE) and coefficient of determination (R^2^) between observations and predictions is reported on the figure.

Figure S1


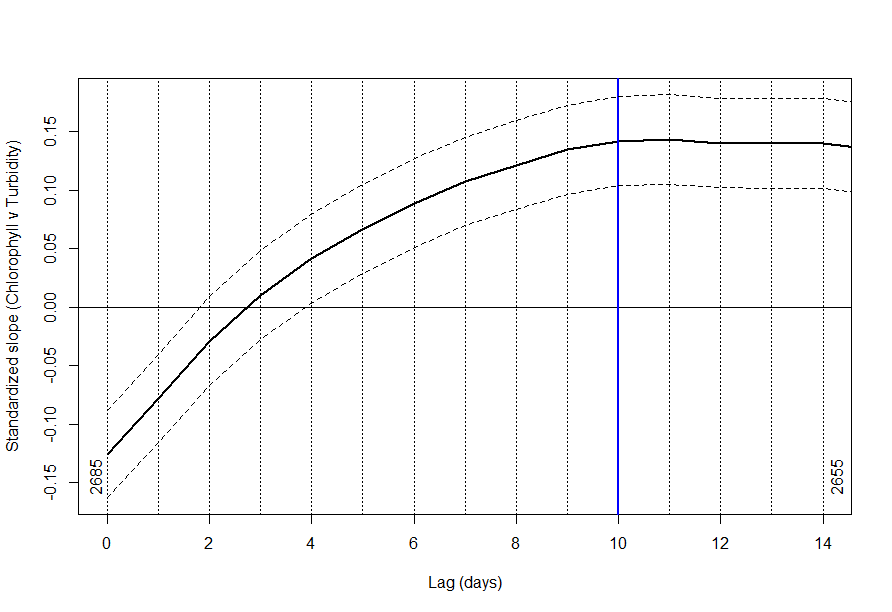


Figure S2


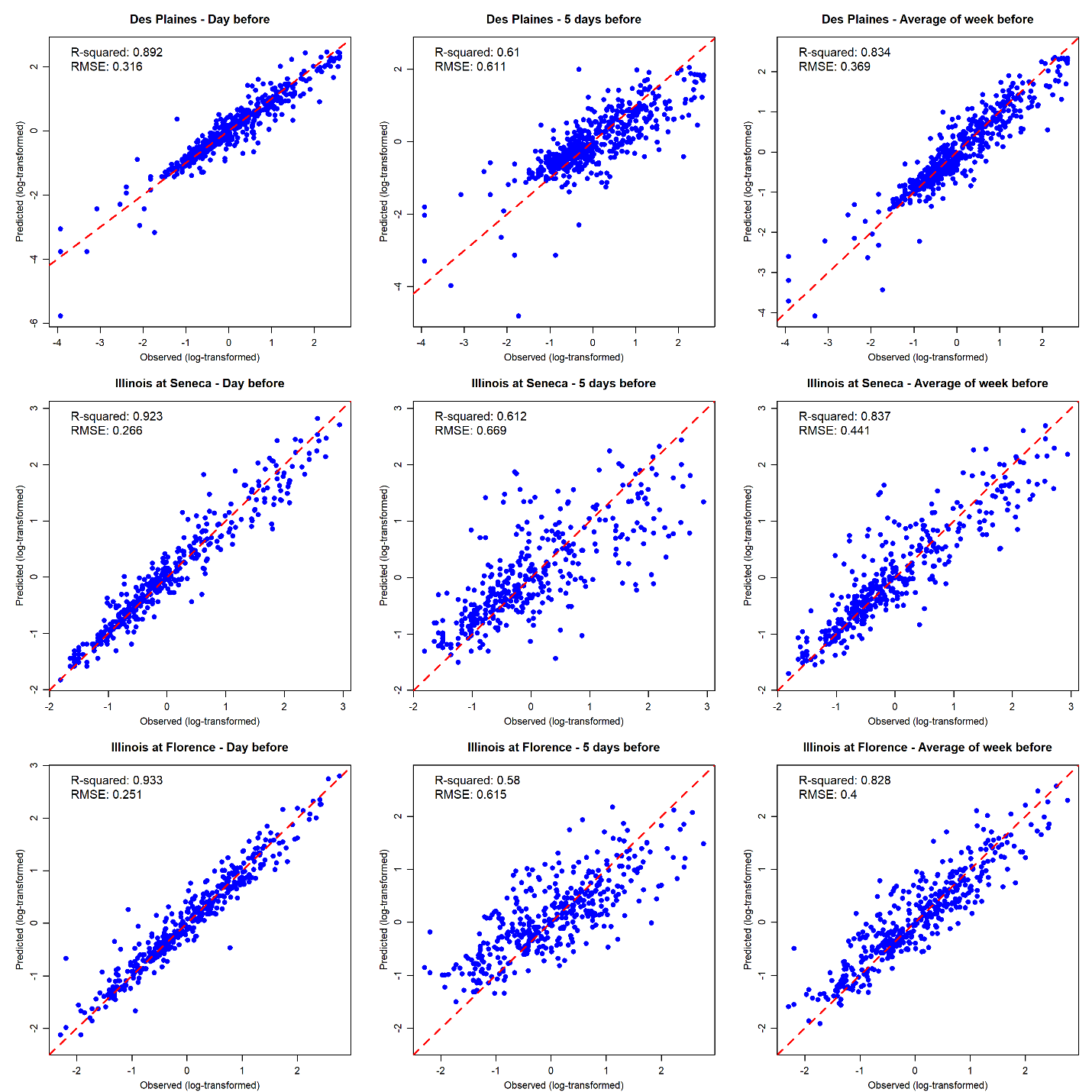

Supplement: Supplementary file 1 — Table S1: Monthly median chlorophyll a concentrations at three sites in the Illinois River basin (IL, USA), 2013–2024 (Platt et al. 2022; U.S. Geological Survey 2024). Monitoring frequency is inconsistent across this time period and remaining analyses are focused on recent years (> 2018). All chlorophyll a concentrations are in units of μg chl a L−1. Figure S1: Relationship between the standardized slope of a model relating chlorophyll a to turbidity and the lag in that model between the day the chlorophyll a was measured and the day the turbidity was measured. For example, the standardized slope of a model relating chlorophyll a measured at day zero to the turbidity measured 10 days before is highlighted in the vertical blue line (this is also the lag day with the greatest slope in this example). Solid line is the standardized slopes and dashed lines are the 95% confidence interval around those standardized slope estimate. The numbers are the maximum and minimum sample sizes (fewer samples are available as the lag times increase). To select the optimal lag time, we used this figure to identify the maximum absolute value of the standardized slope. Figure S2: Comparison of model predictions of chlorophyll a (μg L−1) from univariate linear regression models versus observations that were not included in the original model training. Predictors were log transformed chlorophyll a (μg L−1) concentration the day before, 5 days before or the average of the previous week in the Des Plaines River at Joliet, the Illinois River at Seneca, or the Illinois River at Florence (IL, USA). Dots are observed data, dashed line is the 1:1 line. The root mean square error (RMSE) and coefficient of determination (R 2) between observations and predictions is reported on the figure. [file ECE3-16-e73414-s001.docx]
